# Subarachnoid Hemorrhage Secondary to Forceful Sneeze

**DOI:** 10.1155/2015/896732

**Published:** 2015-01-18

**Authors:** Ali Zohair Nomani, Haris Majid Rajput, Mansoor Iqbal, Zakir Jan, Muhammad Irshad, Mazhar Badshah, Rao Sohail Yasin Khan

**Affiliations:** Department of Neurology, Pakistan Institute of Medical Sciences, Islamabad 44080, Pakistan

## Abstract

Subarachnoid hemorrhage (SAH) is a relatively less common but important neurological condition comprising 5% of all the cerebrovascular accidents. In most populations the reported incidence is 6-7 per 100,000 person-years and one-third of survivors become dependent. It is a serious but potentially treatable cause of neurological morbidity. Multiple authors have identified the most unusual novel associations and triggers of subarachnoid bleeds over the past decade. We herein report a rare case of subarachnoid hemorrhage leading to focal neurological deficit in a middle aged man secondary to forceful sneeze.

## 1. Introduction

Subarachnoid hemorrhage (SAH) is a relatively less common but important neurological condition comprising 5% of all the cerebrovascular accidents. In most populations the reported incidence is 6-7 per 100,000 person-years and one-third of survivors become dependent [1]. It has been found to be around 20 per 100,000 in Finland and Japan [1]. In India and Pakistan, incidence of SAH is still low as compared with that in Japan and the Western world. If early deaths due to SAH however are taken into consideration, the rate would turn out to be as high as 32 per 100,000 [2, 3]. Average case fatality rate has been found to be about 50% in population-based studies, with a trend towards gradual improvement. In India and Pakistan, incidence of SAH is around 5%. About 80% of the cases of SAH are due to rupture of intracranial aneurysms and the rest of 20% are nonaneurysmal [3–6].

Prevention of subarachnoid hemorrhage and indeed diagnosis and therapy requires understanding its pathophysiology. As such, it is important to know which activities can lead to SAH, as it is a serious but potentially treatable cause of neurological morbidity [7–14]. Common etiological associations include intracranial arterial aneurysms, head injuries, and arteriovenous malformations. The triggers that lead to SAH may at times be identifiable while none may be apparent at other times. A few of the recent case reports have suggested novel causes/triggers of subarachnoid hemorrhage [9, 10, 15–23]. We herein report a rare case of intracranial subarachnoid bleed leading to focal neurological deficit in a middle aged man secondary to forceful sneeze. While authors in their review articles, cross-over studies, short communications, and case-control studies have identified sneeze as a potential trigger factor, we did not find any detailed case report describing solely sneeze leading to SAH through thorough database search at Pubmed, Medline, MedlinePlus, PubMed Central, and Pakmedinet. To our knowledge, our case is unique and the first of its kind to be reported from Pakistan.

## 2. Case Presentation

**Figure 1:**
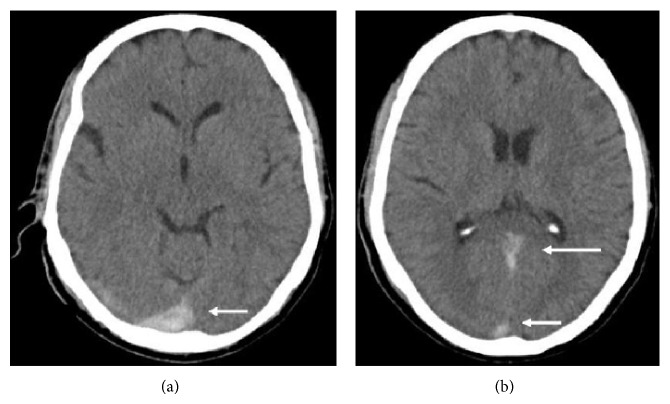
First CT scan brain without contrast showing suspicion of subarachnoid bleed. (a) Hyperdensity involving the posterior most portion of falx cerebri and extending to right (white arrow)-(trickling trail of blood); (b) hyperdensity extending along falx cerebri posteriorly (white arrow long) and marginally to the right (white arrow short).

**Figure 2:**
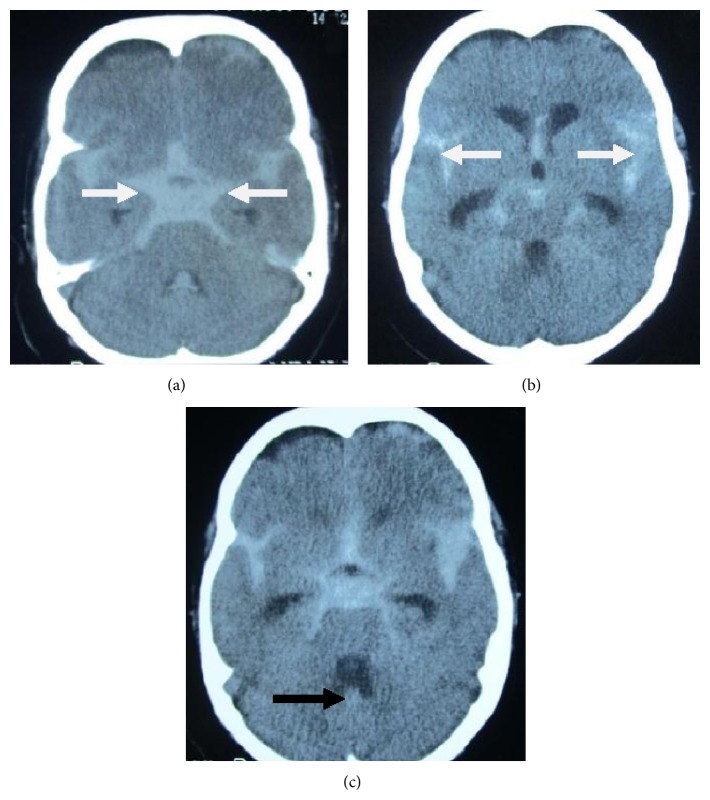
Second CT scan brain without contrast showing clearly visible subarachnoid bleed. (a) Classical star shaped hyperdensity seen filling the subarachnoid space most apparently around the circle of Willis (white arrows); (b) hyperattenuating signal in bilateral sylvian fissures (white arrows); (c) trickle of blood seen extending into the ventricular system (black arrow) (also visible in (a)).

**Figure 3:**
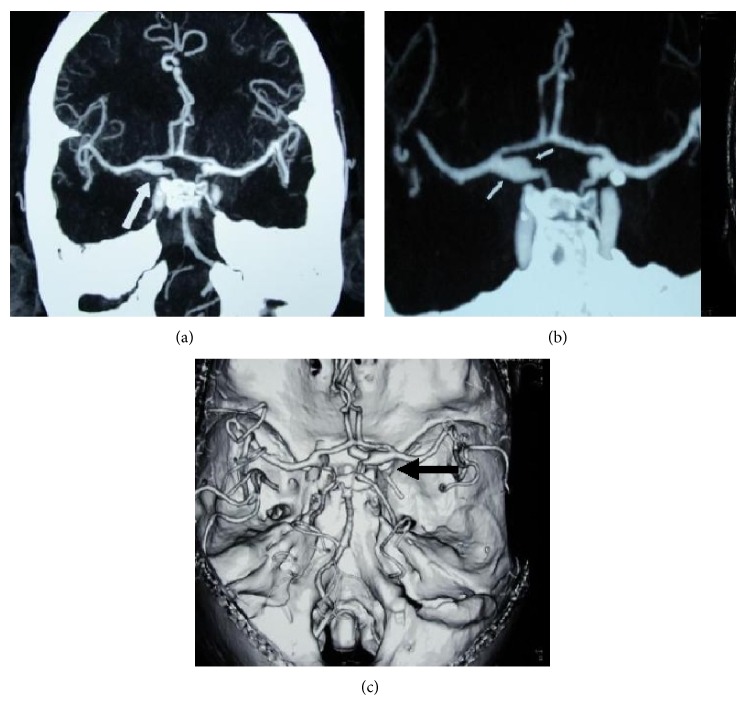
CTA brain showing PCom artery aneurysm. (a) CTA: an aneurysm arising from origin of right posterior communicating artery (PCom) (white arrow); (b) CTA: the aneurysm measured 11 mm in larger dimension with a broad-base/neck of 5 mm and a dome of 3.5 mm (white arrows); (c) vascular reconstruction: right PCom aneurysm on vascular reconstruction imaging (arrowhead) with prominent ipsilateral PCom and correspondingly small/hypoplastic ipsilateral P1 of PCA (posterior cerebral artery).

A 55-year-old gentleman presented to our stroke unit with 2-day history of sudden onset, severe, generalized headache following forceful sneeze. The headache occurred instantaneously following the sneeze while the patient was sitting on a chair watching television. It was the worst headache of patients' life. A consultation with local general practitioner (GP) within 10 minutes of the event revealed normal blood pressure (120/80 mm Hg) and pulse (86/min, regular) but the patient was having neckache and had 3 to 4 episodes of vomiting. This was almost instantaneously followed by right sided numbness and weakness of body while the patient was consulting his GP. Neither did he lose consciousness nor had he fits or fever but he had been irritable and aggressive since then. He was not addicted to any of the elicit or related drugs of abuse. Clinical examination revealed supple neck with right hemiparesis and Broca's aphasia. He was prescribed analgesics and advised Computed Tomography (CT) scan brain. He got his CT done at a local hospital on second day of event after which he was referred to our unit on the same day with suspicion of subarachnoid hemorrhage (Figure 1). We received the patient with above described findings after 46 hours of initial event and he got admitted in our stroke unit. His first CT brain was reviewed by consultant radiologist at our setup who reported the presence of trickling blood along the falx cerebri extending posteriorly and towards the right side into posterior fossa (Figure 1). In the light of suspicion, cerebrospinal fluid (CSF) examination was done to confirm the presence of xanthochromia. A subsequent CT scan brain was done on 4th day of event that revealed clear cut presence of SAH with developing hydrocephalus (Figure 2). A following CT angiography (CTA) revealed aneurysm of the right posterior communicating artery (Figure 3).

The patient was managed conservatively using analgesics, antiemetics, intravenous hydration, nimodipine, and follow-up rehabilitation program as he did not provide consent for neurosurgical intervention. The patient made an uneventful recovery with complete resolution of neurological deficit and was symptom-free on a follow-up visit at 8 weeks.

## 3. Discussion

Subarachnoid hemorrhage is an important cause of neurological deficits. It is mostly associated with ruptured intracranial aneurysms. The triggers responsible for rupture have been studied by many researchers who have pointed out their significance as well as pathophysiology. Reported associations include coagulopathies (either pharmacologically-induced or resulting from systemic diseases), lumbar puncture for diagnostic or anesthesiological purposes, traumatic injuries, aortic coarctation, degenerative vascular diseases, herpes encephalitis, aneurysmal rupture following sexual intercourse, venous hypertension, cortical venous thrombosis, bee sting, epidural blood patch, Takayasu arteritis, brucella meningitis, cranial arterial dissections, contrast agent neurotoxicity, Wegner's granulomatosis, dengue fever, polyarteritis nodosa, moyamoya, and Conn's syndrome [9, 10, 23–34]. While authors have identified sneeze as a potential trigger factor, published cases describing SAH secondary to sneeze have rarely been reported. Matsuda et al. evaluated medical records of 513 patients with aneurysmal subarachnoid hemorrhage. They analyzed the factors precipitating aneurysmal rupture and found that incidence was higher between 6:00 AM to 9:00 AM and 6:00 PM to 9:00 PM when engaging in daily routines such as defecation/micturition, brushing teeth/washing face/dressing, eating/drinking, and taking a bath [35]. Vlak et al. identified independent risk factors for SAH in two of his publications including strenuous activities that resemble valsalva. He identified eight triggers in a crossover study and reported that nose blowing is associated with a relative risk of 2.3 for ACA (anterior communicating artery), 6.6 for ICA (internal carotid artery), 6.2 for MCA (middle cerebral artery), 3.8 for ANT (anterior circulation: combining ACA, MCA, and ICA), and 1.9 for VBA (vertebrobasilar arteries) [36, 37]. While both suggested multiple trigger factors, none reported sneeze in their work. The most plausible explanation of SAH with sneeze relates to the cardiovascular and cerebrovascular effects of the valsalva maneuver on MAP (mean arterial pressure) and ICP (intracranial pressure), thereby altering the transmural pressure gradient across a preexisting aneurysmal wall. This stands true for other triggers as well. Under normal circumstances, cerebral blood flow is maintained constant over a range of cerebral perfusion pressure values by cerebrovascular autoregulation while maintaining a specific narrow range of mean arterial pressure (MAP) [34, 38]. Changes in ICP and blood pressure may alter intracranial blood volume as a result of dilatation or constriction of cerebral blood vessels while volume-pressure changes and brain shifts are taking place (Figure 2). The overall result is a compromised arterial wall at the verge of rupture and aneurysms are the most vulnerable sight for this. The purpose of describing this event getting triggered by such a casual event as sneeze points out to the fact that even the simplest and apparently harmless activities can damage a potentially weak point in the cranial vasculature [38, 39].

## 4. Conclusion

SAH can be triggered by the simplest and apparently harmless activities if there is a potentially weak point in the cranial vasculature. As such, family screening should be duly offered to immediate family members for unruptured, untreated intracranial aneurysms. Moreover, patients and their relatives should be educated about the possible risk factors/triggers for SAH.
